# Misdiagnosis of a Ruptured Ovarian Ectopic Pregnancy as Ovarian Malignancy: Case Report and Literature Review

**DOI:** 10.1155/carm/2904664

**Published:** 2025-09-24

**Authors:** Rebecca Shin, Bridget Neal, Alireza Abidi

**Affiliations:** ^1^Obstetrics and Gynecology, Adventist Health White Memorial, Los Angeles, California, USA; ^2^Medical Education, Rush Medical College, Chicago, Illinois, USA; ^3^Gynecologic Oncology, Kaiser Permanente Riverside Medical Center, Riverside, California, USA

## Abstract

Ruptured ovarian ectopic pregnancy (ROEP) is a rare and life-threatening condition that can increase maternal morbidity and mortality. This case report describes a patient with an atypical presentation that illustrates the diagnostic difficulty of ROEP. Due to overlapping clinical features, it was initially misdiagnosed as an ovarian malignancy. We review relevant clinical symptoms, βHCG trends, and imaging findings that should be evaluated together when considering differential diagnoses. Although most ROEPs are ultimately diagnosed intraoperatively and by histopathology, this case report highlights key diagnostic decision points in the setting of ambiguous clinical and radiologic findings.

## 1. Introduction

An ectopic pregnancy is a rare and potentially life-threatening condition where a fertilized egg grows outside of the uterus. Ectopic pregnancy constitutes 1%-2% of all pregnancies and is a leading cause of maternal morbidity and mortality. Over 90% of ectopic pregnancies occur from implantation within the fallopian tube; however, there are subtypes of ectopic pregnancies that can occur outside the fallopian tube, including but not exclusive to cesarean section scar, abdominal, cervical, interstitial, and ovarian variants [1].

Ovarian ectopic pregnancy is one of the rarest forms of ectopic pregnancy, accounting for 0.5%–3.5% of all cases [2]. While early diagnosis is critical to reduce complications, it remains diagnostically challenging. Although some cases are initially suspected during surgery, definitive diagnosis is typically made via histopathological confirmation [3].

A ruptured ovarian ectopic pregnancy (ROEP) can be misdiagnosed as there are other etiologies with similar presentations, such as a ruptured ovarian cyst or malignancy. In this case report, we present a patient with vague clinical symptoms, abnormal βHCG trend, and ultrasonographic and computed tomography (CT) images leading to our misdiagnosis of ROEP.

## 2. Case Presentation

**Figure 1:**
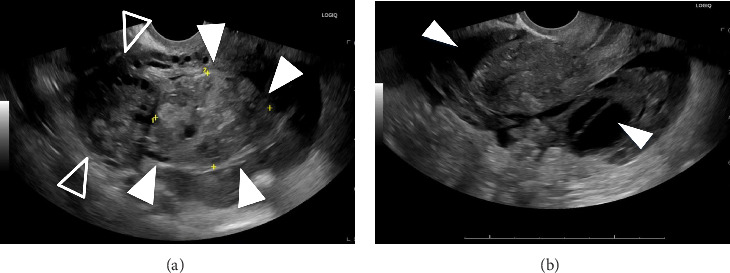
(a) Transvaginal image of 5.0-cm solid and heterogenous mass (indicated with filled arrowheads) abutting the right ovary (indicated by the unfilled arrowheads). (b) Transvaginal image showing free fluid in the cul-de-sac and right adnexa (indicated by the white arrows). Ascites was seen in all quadrants with the largest one in the left upper quadrant (not visualized in image).

**Figure 2:**
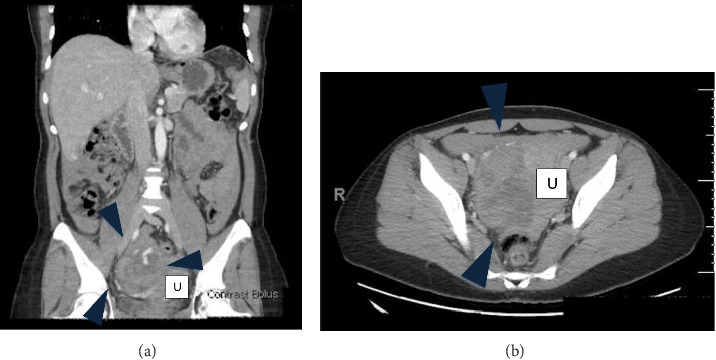
(a) Coronal view of CT showing 8.8 × 6.0 × 8.3-cm right heterogenous density mass with prominent vascularity (indicated by the filled blue arrowheads; U represents the uterus). Small to moderate amount of complex hyperdense free fluid in the abdomen and pelvis. (b) Axial view of CT showing same findings as mentioned above (indicated by the blue arrowhead; U represents the uterus). The mass is also noted to cause mass effect with deviation of the uterus toward the left.

A 33-year-old gravida 3 para 2 who presented to urgent care for a 3-month history of intermittent pelvic pain and pressure. Her last menstrual period was 1 month prior to her presentation, and she was using the NuvaRing for contraception. Vitals and hemoglobin were normal. On physical examination, the patient was mildly tender to palpation throughout the abdomen. Transvaginal ultrasound demonstrated a 5.0-cm solid right ovarian mass, pelvic and abdominal ascites, and simple fluid in the cul-de-sac and right adnexa, with no intrauterine pregnancy (Figures 1(a) and 1(b)). The βHCG level rose from 260 mIU/mL to 301 mIU/mL in a forty-eight-hour period. CT imaging revealed a large 8.8-cm heterogeneous right adnexal mass with peripheral vascularity and complex hyperdense free fluid, raising concern for malignancy (Figures 2(a) and 2(b)). Tumor markers, including carcinoembryonic antigen (CEA) and cancer antigen 125 (CA-125), were within normal limits.

Six days after the initial βHCG draw, the repeat testing showed a decline to 48 mIU/mL. Given the atypical βHCG trend and image findings, a neoplasm of ovarian origin was suspected. The patient was referred to gynecologic oncology for further evaluation.

Two weeks later, the patient underwent exploratory laparotomy, radical excision of pelvic tumor, right salpingo-oophorectomy, left ovarian cystectomy, and dilation and curettage with concern for a germ cell or sex cord tumor. Intraoperative findings included a large solid right adnexal mass with dense adhesions involving the bladder peritoneum, cul-de-sac, uterus, and sigmoid colon. A small implant was noted on the small bowel. Dilation and curettage of the endometrial cavity were unremarkable.

Intraoperative frozen section was remarkable for a benign hemorrhagic cyst. Final pathology reported a large hemorrhagic cavity containing scattered degenerating chorionic villi adjacent to the ovary and fallopian tube, compatible with an ectopic pregnancy. Implants removed from the uterus, sigmoid colon, small bowel, bladder, right pelvic peritoneum, and cul-de-sac were remarkable for fibrotic tissue with mild chronic inflammation, indicating a chronic ROEP. The βHCG postoperatively down trended to 2 mIU/mL. The patient recovered well and was discharged on the same day. She remained stable at her postoperative follow-up.

## 3. Discussion

Ovarian ectopic pregnancy is a rare and potentially life-threatening condition, typically presenting with acute abdominal pain and/or vaginal bleeding. Most ovarian ectopic pregnancies present with hemodynamic instability due to hemoperitoneum [2]. In contrast, our patient was hemodynamically stable with a prolonged history of pelvic discomfort, highlighting the diagnostic variability of ROEP. The atypical βHCG trend and nonspecific imaging findings led to initial suspicion of ovarian malignancy and subsequent misdiagnosis.

Serum βHCG levels in ectopic pregnancy can vary widely and are not always predictive of rupture or disease severity. In a retrospective review by Eisaman et al., 50.4% of ectopic pregnancies had βHCG levels less than 1500 mIU/mL and 8.5% were less than 100 mIU/mL. Furthermore, βHCG levels did not correlate with the presence or size of an ectopic pregnancy [4]. Similarly, Dimarchi et al. reported that approximately 10% of ectopic pregnancies with βHCG level less than 100 mIU/mL had ruptured, with 7% of those ruptures occurring at levels below 100 mIU/mL [5]. Although it is theoretically possible that infarction or necrosis of an ovarian tumor could cause a sudden decline in βHCG levels, this phenomenon is not well-documented in the literature. In this case, the rapid decrease in βHCG was more consistent with a spontaneously resolving ruptured ectopic pregnancy. This underscores the importance of interpreting βHCG levels in the context of clinical presentation and imaging rather than as a sole diagnostic indicator.

Imaging findings in ROEP can mimic those of other adnexal pathologies, including ruptured corpus luteum cyst, hemorrhagic ovarian cyst, and tubal ectopic pregnancy. In our patient, ultrasound revealed a solid mass with peripheral vascularity and free fluid, without the classic “ring of fire” sign. The presence of an ovarian cortex with follicles around the mass demonstrated the ovarian origin. However, vascularity on imaging is not specific to ROEP and can also be seen in neoplastic lesions. Notably, the patient's presentation lacked the acute hemodynamic instability typically associated with rupture, making the timing of rupture difficult to ascertain.

Free fluid in the pelvis is a nonspecific finding and can be present in both hemorrhagic cyst rupture and ROEP. In some cases, the presence of trophoblastic tissue may induce vascular malformations, further complicating differentiation from ovarian malignancies [6]. Ren et al. advocate for consultation with experienced sonographers in suspected ovarian ectopic pregnancies, although the role of ultrasound in diagnosing ROEP specifically is still underexplored and warrants further research [7].

The accuracy of CT for ROEP remains limited as well. It is more often used to evaluate alternative causes of abdominal pain or hemoperitoneum. As seen in this case and others, such as the series by Shin et al., ectopic pregnancies may appear as heterogenous enhancing adnexal masses with significant intraperitoneal hemorrhage on CT [8]. The literature suggests the need for further investigation to understand how to better differentiate between ROEP, ruptured ovarian cyst, and ruptured tumor. Thus, CT or MRI may still aid in preoperative planning for patients with stable vital signs, particularly when malignancy is part of the differential.

There are a few cases in the literature that have misdiagnosed a ruptured ovarian cyst or ectopic pregnancy for an ovarian malignancy. Savelli et al. described two patients with failed tubal ectopic pregnancies, in which transvaginal ultrasound demonstrated hypervascular solid adnexal masses that were initially mistaken for ovarian malignancies. The authors suggest that vascular malformations can be induced by trophoblastic tissue, which can lead to the hypervascularized appearance on sonographic imaging [9]. The diagnoses were ultimately confirmed after laparoscopy and histology. In another case report, Qing et al. reported a ruptured primary ovarian pregnancy initially mistaken as an ovarian malignancy due to a complex adnexal mass and significant hemoperitoneum. The true diagnosis was also made intraoperatively and confirmed histologically, highlighting how ovarian ectopic pregnancy can closely mimic an ovarian cancer and cause preoperative diagnostic confusion [10].

In the setting of an abnormal βHCG trend and large heterogeneous mass, ovarian malignancy must remain a key consideration. Differential diagnoses include germ cell tumors, gestational trophoblastic disease, and choriocarcinoma—all of which may present with elevated βHCG and acute abdomen due to rupture or torsion [11, 12]. There are documented instances of malignancies that mimic ovarian ectopic pregnancies. For example, Kucera et al. reported on an apparent ectopic pregnancy that was found to be a germ cell tumor, which was discovered intraoperatively [13]. While Heo et al. and Chen et al. reported nongestational ovarian choriocarcinomas presenting with hemoperitoneum and elevated βHCG levels in a 12- and 23-years old, respectively [14, 15].

These cases, including ours, illustrate the importance of keeping malignancy on the differential when evaluating adnexal masses with atypical features, such as size larger than 10 cm, solid or papillary components, irregular septations, internal vascularity, or associated ascites. Clinical correlation and detailed imaging assessment are essential, yet differentiation can be challenging when a ruptured ectopic pregnancy presents chronically with features that can closely resemble those of ovarian malignancy. To minimize the risk of misdiagnosis, a broad differential diagnosis should be maintained when evaluating adnexal masses, especially in reproductive-age women. Preoperative counseling and intraoperative preparedness can help mitigate the risk of incorrect surgical management. Ultimately, surgical exploration and histopathological examination remain the gold standard for diagnosing ROEP.

## 4. Conclusion

ROEP is a rare and potentially life-threatening condition. Preoperative diagnosis remains a challenge, especially when patients have vague symptoms and findings on imaging. Review of the βHCG trend and diagnostic imaging should be jointly used to determine a list of differential diagnoses. The utilization of ultrasound and CT remains to be an area of improvement to better differentiate ROEP and etiologies with similar presentations. Lastly, a gynecologic oncology referral should always be considered in nonspecific cases to provide proper perioperative management.

## Data Availability

All data generated or analyzed in this case report are included within the published article. No additional datasets were generated or analyzed.
